# Synthesis and Olfactory Evaluation of Bulky Moiety-Modified Analogues to the Sandalwood Odorant Polysantol^®^

**DOI:** 10.3390/molecules14082780

**Published:** 2009-07-28

**Authors:** Laura Chapado, Pablo J. Linares-Palomino, Manuel Nogueras, Adolfo Sánchez, Joaquín Altarejos

**Affiliations:** Departamento de Química Inorgánica y Orgánica, Facultad de Ciencias Experimentales, Universidad de Jaén, 23071 Jaén, Spain

**Keywords:** sandalwood odorants, Polysantol^®^ analogues, nopol derivative, odour evaluation

## Abstract

Five new bulky moiety-modified analogues of the sandalwood odorant Polysantol^®^ have been synthesized by aldol condensation of appropriate aldehydes with butanone, deconjugative α-methylation of the resulting α,β-unsaturated ketones, and reduction of the corresponding β,γ-unsaturated ketones. The final compounds were evaluated organoleptically and one of them seemed to be of special interest for its natural sandalwood scent.

## 1. Introduction

**Figure 1:**
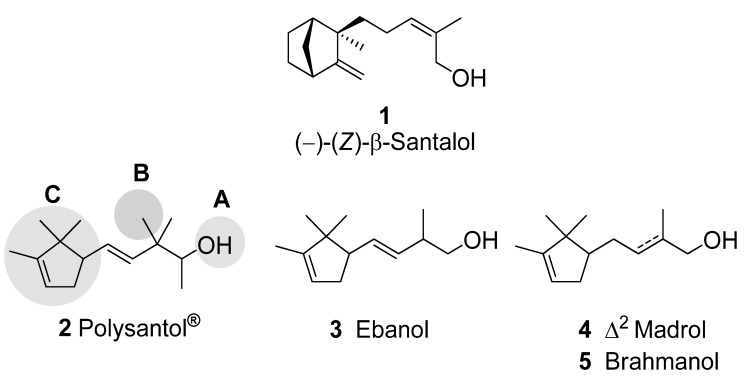
β-Santalol and odorants **2**–**5** derived from campholenic aldehyde.

(–)-(*Z*)-β-Santalol (**1**), the main constituent of natural sandalwood oil, is an odour compound with typical sandalwood fragrance and is described as warm-woody, creamy and sweet with an animalic tonality [1,2]. It consists of a bulky bicyclic moiety separated from the hydroxyl group by an unsaturated 5 C-atoms spacer [3]. The best synthetic substitutes for this noble perfumery raw material are a series of trimethylcyclopentenyl alkenols, such as **2−5** [4,5],derived from campholenic aldehyde. The structural similarities between β-santalol and these substitutes, regarding the bulky lipophile, the spacer and the osmophoric polar hydroxyl group, seems to be clear (Figure 1), Polysantol^®^ (**2**) being the most expensive and appreciated by perfumers [3]. The structure–odour properties of this compound and a series of derivatives have been studied [6,7,8]. The structure of olfactory receptors and the corresponding mechanism of interaction between receptor proteins and odour molecules, rewarded by the 2004 Nobel Prize [9,10], are still little known. Therefore, the determination of essential structural elements responsible for the sandalwood-type sensation can be only performed by molecular similarity studies within a series of sandalwood odour compounds and structurally similar, but odourless, molecules. As is well known [3,11], three subunits are important for the sandalwood odour impression (Figure 1), which correspond to the hydroxyl group (A), a lipophilic substituent (B) in the neighbourhood of this hydroxyl group, and a bulky rigid hydrophobic moiety (C). This set of structural features constitutes the sandalwood olfactophore. In this way, some fragrance chemists assumed that the vicinity of the osmophore must be crucial for the odour and this flexible spacer became the main object of structure–odour sandalwood studies [12]. On the other hand, the analysis of structure–odour relationship (SOR) data allowed to postulate that the geometry of the immediate proximity of the osmophoric hydroxyl group tolerates less variations than the orientation of the more distant lipophilic bulky group [3]. For that reason the bulky moiety of the trimethylcyclopentenyl group in campholenal derivatives **2−5** has been replaced by structures of similar steric bulk (**6** [13], **7** [14], **8** [15], **9** [16], **10** [17], **11** [18]).

As a continuation of our previous studies on the synthesis of odorants [19,20,21],we have developed a collection of several substitutes of sandalwood scent [22,23,24]. As other authors have done [7,8,25], we have studied the influence of the global shape of the hydrophobic moiety C, and for the refinement of the olfactophore model on compounds structurally similar to Polysantol^®^, five new compounds **33**−**37** (Figure 3) have been synthesized for this work and their odour evaluated. These molecules have been obtained from the aldehydes **12**−**15** and **18**, respectively, through a straightforward process involving the aldol condensation of each starting aldehyde with butanone, the deconjugative α-methylation of the respective enones and the reduction of the corresponding β,γ-unsaturated ketone to yield every alcohol analogue to the odorant Polysantol^®^ (see Scheme 2 below).

**Figure 2:**
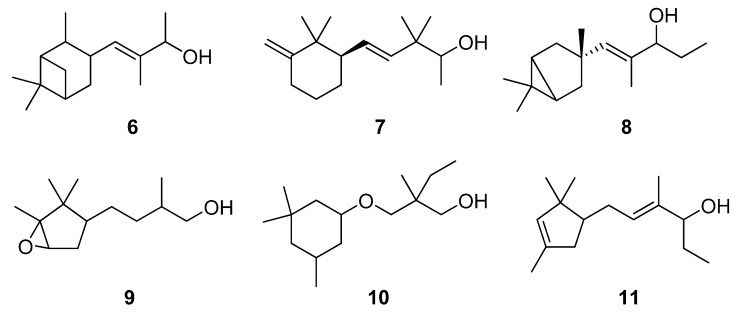
Other sandalwood-type odorants with different bulky moieties.

**Figure 3:**
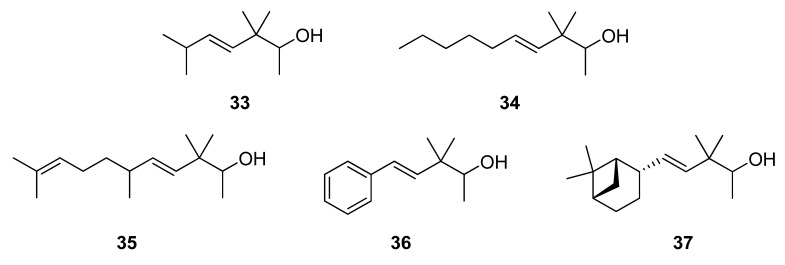
The target analogues of the sandalwood odorant Polysantol^®^.

## 2. Results and Discussion

### 2.1. Synthesis

As starting materials for the synthesis of alcohols **33**–**37**, the commercially available acyclic isovaleraldehyde (**12**), heptanal (**13**), citronellal (**14**), and the cyclic phenylacetaldehyde (**15**) and (1*R*)-(–)-nopol (**16**) [26] have been chosen. The latter was previously transformed into (1*S*,2*S*,5*S*)-dihydronopal (**18**), in a two-step process, by stereoselective heterogeneous hydrogenation using platinum oxide as catalyst [19,27] and subsequent oxidation of the primary alcohol to an aldehyde with pyridinium dichromate (PDC) [29].

#### 2.1.1. Conversion of nopol (**16**) into dihydronopal (**18**)

**Scheme 1:**
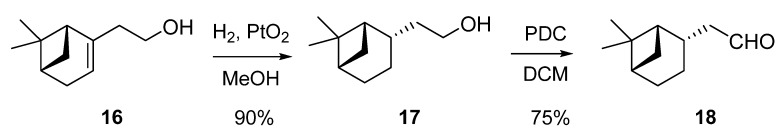
Selective hydrogenation of nopol and oxidation of *cis*-dihydronopol under mild conditions.

According to the findings of Heitmann and Mätzel [27], the use of *Adams ’* catalyst in methanol with a low hydrogen pressure allowed us to obtain *cis*-dihydronopol (**17**) [28,29] in good yield (90%) and with high diastereoselectivity. Therefore, the substituent in the 2 position is *cis* with respect to the *gem*-dimethyl bridge in **17** (Scheme 1).

**Figure 4:**
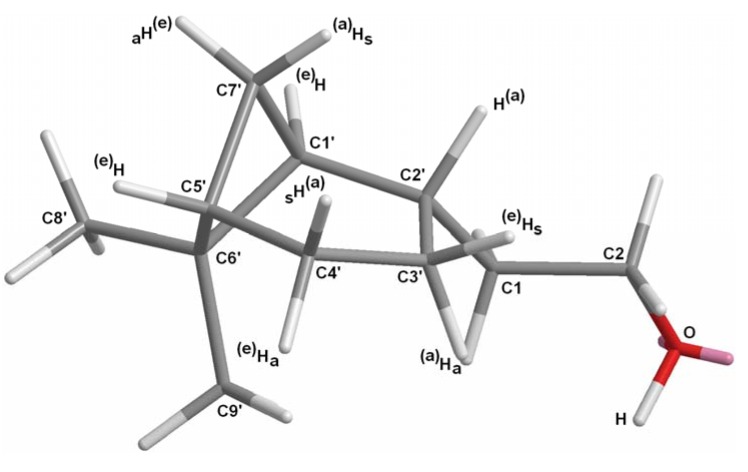
(1*S*,2*S,*5*S*)-2-(6,6-dimethylbicyclo[3.1.1]hept-2-yl)ethanol (**17**).

The structure of this compound was assigned by standard spectroscopic analytical techniques (IR, MS, ^1^H-NMR, ^13^C-NMR, 2D NMR). The bicyclic system of the [3.1.1]hept-2-yl group presents several particularly troublesome spectroscopic problems, since its structural complexity leads to strong couplings among nuclei, resulting in severe spectral overlap. Nevertheless, some unambiguous conclusions may be obtained. Thus, the final assignment of the chemical shifts and coupling constants of all compounds of this series (**17**, **18**, **27**, **32**, **37**) are given taking into account the *syn* (s) and *anti* (a) protons, which correspond to equatorial or axial protons of a cyclohexane moiety (Figure 4).

The ^1^H-NMR of **17** has undoubtedly lost the characteristic tt signal of the olefinic methyne H-3’ seen in **16**. In **17** the new methyne H-2’ appears as a ddq (δ 2.12, *J*_2’-1’_=2.0 Hz, *J*_2’-3’a-2_=7.1 Hz, *J*_2’‑3’s_=11.0 Hz). The coupling constants between H2’–H3’a and H2’–H3’s could be consistent with dihedral angles H2’–C–C–H3’a and H2’–C–C–H3’s of *ca.* 124º and 10º, respectively, according to the *Karplus* equation [31,32]. These observations not only provide evidence for the necessary axial position of the new H-2’ proton, but also confirm the postulate that the cyclohexane ring is flattened, confirming the outcome of the diastereoselective hydrogenation. The difference between the protons of the CH_2_-7’ methylene bridge is characteristic of these type of bicyclic [3.1.1]heptane skeletons in a bridged-chair or bridged-boat conformation. Thus, proton 7’a appears as a ddt (δ 2.33, *J*_7’a-7’s_=9.3 Hz, *J*_7’a-1’-5’_=6.2 Hz, *J*_7’a-4’a_=2.0 Hz) on the basis of the geminal coupling with H-7’s, similar couplings with H-1’ and H-5’ and the W long-range coupling with H-4’a. However, the 7’s proton (δ 0.90) appears as a d because of a single geminal coupling. The different resonance signals of the two methyl groups on C-6’, due to the magnetic anisotropy of the cyclobutane ring, is also characteristic of this skeleton. Hence, Me-8’ (equatorial) always appears *ca* 0.4 ppm deshielded respect to Me-9’ (axial) in 2-α-pinene derivatives (such as **16**). Nevertheless, in 2-α*H*-pinane derivatives (such as **17**) the less rigid geometry compared to the saturated system produces an appreciable change in the resonance position of the equatorial and axial methyl groups on C-6’, the Δδ between them being now *ca.* 0.1 ppm. The highly overlapped region of δ 1.80−2.00, corresponding to the 1’, 3’s, 4’a, 4’s and 5’ protons was too poorly separated for determination of coupling constants. However, the chemical shifts of such protons were obtained from the 2D NMR shift correlations (HSQC, HMBC, COSY and NOESY). In addition, homodecoupling experiments were also performed to obtain some coupling constants. In general, the relationship δHa < δHe is valid, except for H-4, where 4a (an equatorial proton) resonates at higher field than 4s (an axial proton), which is in accordance with the finding for protons attached to a cyclohexane ring [31]. The conversion of dihydronopol (**17**) into dihydronopal (**18**) [33] was performed by reaction with PDC under standard conditions affording **18** in a 75% yield (Scheme 1).

#### 2.1.2. Aldol condensation of the aldehydes **12**−**15** and **18** with butanone to give the α,β-unsaturated ketones **19**, **20**, **22**, **24** and **27**

**Scheme 2:**
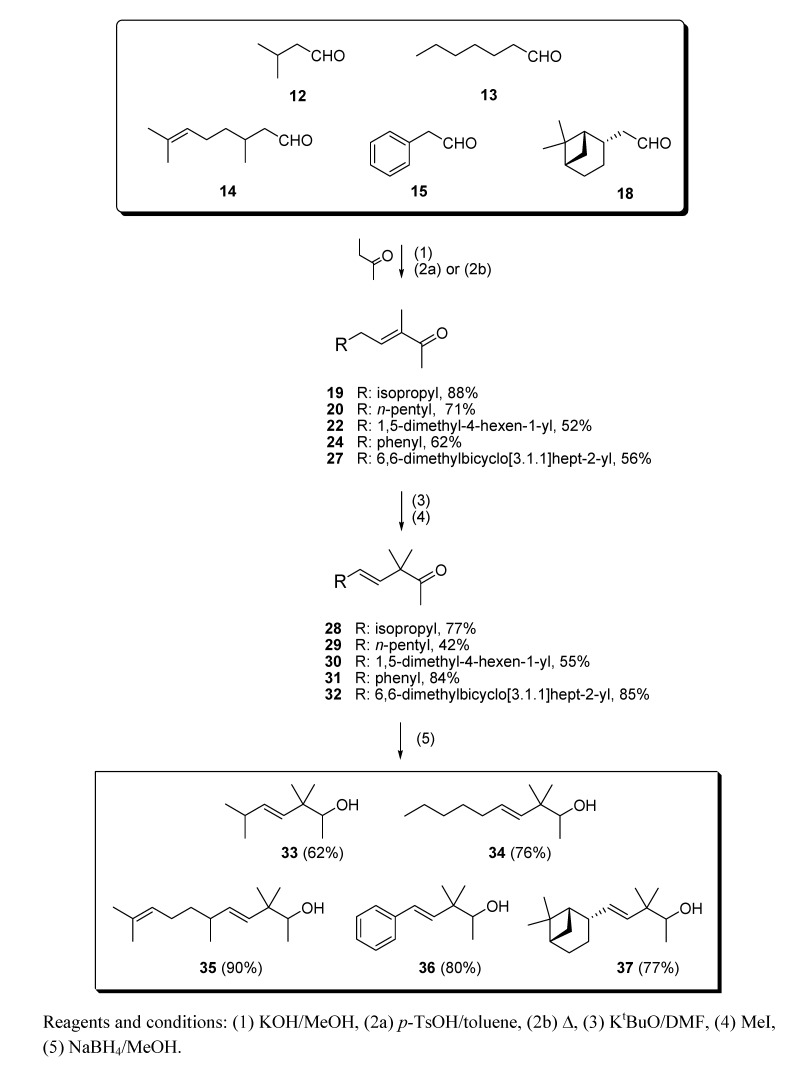
Syntheses of **33**-**37**.

The starting aldehydes **12**−**15** and **18** were reacted with butanone by aldol condensation. Thus, isovaleraldehyde (**12**) yielded the α,β-unsaturated ketone **19** using potassium hydroxide as catalyst [34] (Scheme 2). The intermediate β-hydroxyketone was directly dehydrated by azeotropic distillation in dry toluene and *p*-toluenesulfonic acid [23,35]. The crude **19** obtained was purified by flash chromatography to afford pure **19** in 88% yields.

For the synthesis of **20** [36], heptanal (**13**) was reacted in a similar manner; aldol reaction with butanone followed by *p*-toluenesulfonic acid-assisted dehydration. The α,β-unsaturated ketone **20** was obtained in 71% yield [38]. The spectroscopic properties of **20** and **19** are alike with respect to the synthon C1–C5, and regarding **21**, the aldol self-condensation by-product derived from **13,** the NMR data agree with those already reported [39].

When citronellal (**14**) was used as starting aldehyde, the aldol condensation with butanone to obtain the α,β-unsaturated ketone **22** was performed using a basic thermal dehydration instead the acid dehydration [40]. This was necessary because although all the aldol condensation attempts *via* acid dehydration led to the desired **22**, it immediately underwent an intramolecular *Michael*-type reaction that led first to a six-membered ring closure, and then, after subsequent capture of the emerging tertiary cation by the enol oxygen, to a second ring closure [41].

**Scheme 3:**
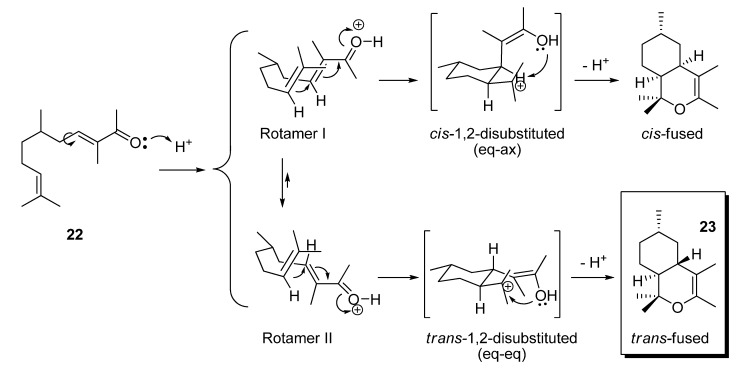
Intramolecular Michael-type ring closure reactions followed by *cis* and *trans* cation intermediates capture by the enol oxygen toward *cis* and *trans* (**23**) hexahydro-1*H*-isochromenes.

As in the case described by Sasaki [41], we only obtained the *trans*-fused hexahydroisochromene **23**. This stereoselective nonsynchronous bicyclization may be rationalized taking into account the rule for 1,2-disubstituted cyclohexane compounds. According to that, the thermodynamically more stable conformation is that with more alkyl groups adopting the equatorial position. As displayed in Scheme 3, rotamer I leads to a *cis*-1,2-disubstituted cyclohexane (one axial and the other equatorial) whereas rotamer II leads to a *trans*-1,2-disubstituted cyclohexane with both groups in equatorial position. In addition to this energetic argument, it seems that in a *trans*-1,2-disubstituted eq–eq conformation the oxygen of the enol function and the carbocation centre are likely closer for the second cyclization than that in a *cis*-1,2-disubstituted eq–ax conformation.

The structure of compound **23** was assigned by standard spectroscopic techniques (IR, MS, ^1^H- NMR, ^13^C-NMR, 2D NMR). It is worth underscoring some details of its ^1^H NMR like the upshielded resonance of H-5ax as a q (δ 0.59, *J*_5ax-5eq-4a-6_=12 Hz), due to the magnetic anisotropy of the double bond (Δ^3^). Furthermore, the chemical shift assigned to H-4a (δ 1.55–1.68) seems to be a br t, where the highest coupling constant is *ca.* 12 Hz. This is sufficiently consistent with both dihedral angles H4a–C–C–H8a and H4a–C–C–H5ax of 180º, what are the corresponding angles of a *trans*-fused bicyclic system in which the two hydrogen atoms of the junction carbons are both axial.

**Scheme 4:**
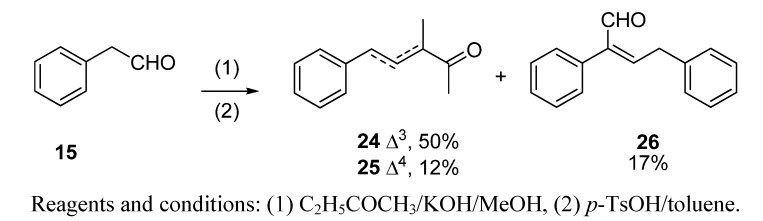
Synthesis of **24**-**26**.

The aldol reaction of phenylacetaldehyde (**15**) with butanone, followed by acid dehydration, yielded the α,β-unsaturated ketone **24** [42], along with the side product **25** [43,45], its positional isomer, and **26**, the aldol-type self condensation of phenylacetaldehyde (Scheme 4). Regarding compound **26**, the spectroscopic data agree with those already reported [44].

In the synthesis of the α,β-unsaturated ketone **27**, *cis*-dihydronopal (**18**) was reacted in a similar way – aldol reaction with butanone followed by acid catalyzed dehydration. The crude obtained was purified by flash chromatography to afford **27** in 56% yields. With respect to the spectroscopic data of **27**, no dramatic changes occur either the C1–C5 moiety, with respect to the analogues **19**, **20**, **22** and **24**, or the 2-α*H*-pinane moiety, with respect to the precursors **17** and **18**. A C’-2 epimer of **27** was prepared by Mookherjee and co-workers and described as possessing a powerful sandalwood aroma with urine [47], sweet and floral undertones. In this patent the inventors claimed its use for enhancing the aroma or taste of smoking tobacco and tobacco articles.

#### 2.1.3. Deconjugative α-methylation of the α, β-unsaturated ketones **19**, **20**, **22**, **24** and **27** to give the β, γ-unsaturated ketones **28–32**

The ketones **19**, **20**, **22**, **24** (+**25**) and **27** could be converted into the corresponding β,γ-unsaturated ketones **28–32** by a deconjugative α-methylation reaction [23,48,49]. This procedure relies on the initial formation of an enolate, using a slight stoichiometric excess of potassium *t*-butoxide, followed by the methylation of the resulting ion under conditions that provided the kinetically favoured product in excess over the thermodynamically favoured product. A ten molar excess of cooled iodomethane was added quickly over the cooled (0 °C) solution of the referred enolate in DMF. The configuration about the double bond in compounds **28**-**32** was *E*, as indicated, and the procedure provided those five new enones, which after chromatographic purification yielded pure compounds **28** (77%), **29** (42%), **30** (55%), **31** (84%) [50] and **32** (85%).

#### 2.1.4. Reduction of the β,γ-unsaturated ketones **28–32** with NaBH4 to give the alcohols **33**–**37**

Finally, β,γ-unsaturated ketones **28–32** could be converted into the corresponding homoallylic alcohols by reducing the carbonyl group with sodium borohydride under standard conditions. Although other reducing agents were also tested [51], a mixture of sodium borohydride in methanol was preferred because of economic considerations and easy handling. As expected, alcohols **33**–**37** were obtained without any stereoselectivity on the new stereocentre C-2. After chromatographic purification the target analogues to Polysantol^®^ were obtained in good yields: **33** (62%), **34** (76%), **35** (90%), **36** (80%) and **37** (77%).

### 2.2. Odour evaluation

The independent odour evaluation of each bulky moiety-modified Polysantol^® ^analogue **33**–**37** (each over 97% pure according to GC) was carried out by a group of perfumers using two different protocols: (a) at three times from impregnated blotting paper strips (see Section 3.6) (Table 1), (b) after injecting them, separately, onto a GC fitted with sniffing port (Table 2).

Thus, the profile of the (*E*)-3,3-dimethyl-5-((1*S*,2*S*,5*S*)-6,6-dimethylbicyclo[3.1.1]hept-2-yl)pent-4-en-2-ol (**37**) was identified as the most interesting and promising of the series because of it is full of qualities and it directly emulates the natural sandalwood odour instead of that of synthetic Polysantol^®^. For that reason, this compound has recently been claimed as a potential useful odorant [24]. Furthermore, it is noteworthy that **34** and **35** display fairly good behaviour as woody and sandalwood odorants, a fact that supports the hypothesis that structurally rigid molecules interact with a smaller number of olfactory receptor proteins than fairly flexible molecules, which can be assumed to interact with the proteins involved in a more complex manner [52].

**Table 1:** Odour evaluation of alcohols **33**–**37** from impregnated blotting paper strips.

Compounds	Odour		
	Top notes	Heart notes	Base notes
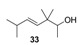	Turpentine, varnish and woody with oriental bottom, patchouli, humid tar, smoky and earthy	Nuance of woody, slightly damp in a mixture between the scent of fresh wood and antique furniture	Slightly eastern woody, not very intense
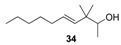	Greasy, citrus and earthy-green notes suitable with a moist mushroom scent	Citronellic-type of citrus odour with weak woody	Green and grassy resembling to freshly cut stalk of palms
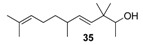	Phenolic, dump, cresolic, milky and sandela flavour	weak citrus odour	almost odourless
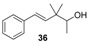	Cresolic, citrus on citronella-type odours, green, phenolic and slightly exotic oriental odour	Slight woody note with flowery touch of roses, but less intense	Imperceptible odour (nearly odourless)
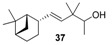	Solvent and woody note, sandalwood-type alike to the essential oil	Sandal and sandela scent	Reminiscence of sandalwood odour with saffron touch

**Table 2:** Odour evaluation of alcohols **33**–**37** using a GC fitted with sniffing port.

Compounds	Odour
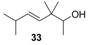	Borneol, balsamic, camphoraceus woody notes, but not sandalwood. Also fencholic, slightly valerianic with a note which remembers to wet mossy forest soil at the end.
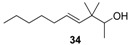	Woody notes with dryness and amber nuances Iso E Super-type. Also fatty, green, floral and soapy notes, with an animalic and valerianic tone at the end.
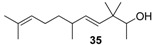	Very clean and natural sandalwood note, Polysantol-type and as intense as this. The woody bouquet is harmonized with amber, balsamic, animalic, sweet, green and a slightly cresolic background.
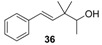	Woody and mild sandalwood scent. It is also slightly greasy with burn, moist and green nuances, and a reminiscent of citrus fruits at the end.
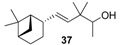	Very clean, intense and rounded sandalwood note, more natural scent than Polysantol-type. It is also woody, vetiver, green in mossy-type, with animal and vanilla notes at the end.

## 3. Experimental

### 3.1. General

Reactions were monitored by gas chromatography (GC) on a Varian CP-3800 gas chromatograph fitted with a methyl silicone (CP-Sil 8 CB) capillary column (30 m × 0.25 mm × 0.25 μm); carrier gas: He; flow rate: 1 mL/min; oven temperature program: 50−290 °C at a rate of 8 °C/min; injector temperature: 250 °C; flame ionization detector temperature: 300 °C; retention times (*t*_R_) are expressed in minutes. The reaction products were purified by conventional column chromatography (Merck silica gel 60, 70−230 mesh) or by flash chromatography (Scharlab silica gel 60, 230−400 mesh), in both cases using appropriate mixtures of hexane and Et_2_O. ^1^H-NMR spectra were recorded on a Bruker DPX 300 spectrometer (300 MHz, CDCl_3_, TMS) and a Bruker DPX 400 (400 MHz) spectrometer. Chemical shift values are reported in parts per million (ppm, δ scale) and coupling constants (*J*) are in hertz (Hz). All described coupling constants refer to a three-bond coupling distance (^3^*J*). ^13^C-NMR spectra were recorded on the same instruments (75 or 100 MHz, CDCl_3_, TMS). Chemical shifts are also reported in ppm and carbon substitution degrees were established by DEPT multipulse sequence. 2D NMR experiments (DQF-COSY, HSQC, HMBC, NOESY) were carried out for all compounds of dihydronopol series (**17**, **18**, **27**, **32**, **37**) and for the isochromene **23**, on the same instrument. Infrared (IR) spectra were recorder on a FT-IR Perkin-Elmer 1760X spectrometer using a thin film between KBr plates (neat). Mass spectra (MS) were obtained in all cases by GC− MS analysis carried out on a Hewlett-Packard 5990 A II gas chromatograph coupled to a Hewlett-Packard 5989B mass spectrometer using the electron impact (EI) ionization method (70 eV); the parameters for the GC unit were the same as those described previously for the GC analyses. High-resolution mass spectra (HRMS) were obtained on a trisector EBE Waters Micromass AutoSpect NT spectrometer using EI (70 eV).

### 3.2. Starting materials

Isovaleraldehyde (3-methylbutanal, **12**): Aldrich, 97% (GC); *t*_R_ 2.26. Heptanal (**13**): Aldrich, 95% (GC); *t*_R_ 6.32. (±)-Citronellal (3,7-dimethyl-6-octenal, **14**): Fluka, 90% (GC); *t*_R_ 16.20. 2-Phenyl-acetaldehyde (**15**): Fluka, 50% solution in diethylphthalate. To separate 2-phenylacetaldehyde (195 °C, 1 atm), *t*_R_ 11.40, from the non-volatile diethylphthalate (295 °C, 1 atm) a vacuum distillation was performed. (1*S*,2*S*,5*S*)-Dihydronopal (**18**) was obtained, as described below, from (1*R*)-(–)-nopol [(1*R*)-2-(6,6-dimethylbicyclo[3.1.1]-2-hepten-2-yl)ethanol, **16**): Aldrich, 98% (GC); [α]^25^_D_ = –31.8 (*c* 1.15, MeOH); *t*_R_ 21.60.

*(1S,2S,5S)-2-(6,6-Dimethylbicyclo[3.1.1]hept-2-yl)ethanol* (**17**): a solution of the alkene **16** (250 mg, 1.5 mmol) in absolute MeOH (12.5 mL) was hydrogenated over PtO_2_ (23 mg) under the low-pressure of a H_2_ gas filled balloon for 90 min. At this time the GC analysis indicated the hydrogenation was complete and the catalyst being filtered off and washed with MeOH. The solvent was evaporated under reduced pressure to afford **17** (227 mg, 90%) as a colourless oil; [α]^25^_D_ = –22.1(*c* 1.25, MeOH); *t*_R_ 24.65; IR (ν, cm^-1^): 3332 (OH), 2907 (cyclohexane), 1383 and 1366 (C(CH_3_)_2_); MS (*m/z*, %): 168 (M^+^, 1), 150 (M^+^−H_2_O, 1), 135 (M^+^−H_2_O−Me, 5), 123 (M^+^−C_2_H_4_OH, 14), 107 (C_8_H_11_^+^, 33); ^1^H-NMR (400 MHz): δ 3.65 (dt, *J*_1A-1B_=10.1, *J*_1A-2A-2B_=7.0, 1H, H-1A), 3.62 (dt, *J*_1A-1B_=10.1, *J*_1A-2A-2B_=7.0, 1H, H-1B), 1.68 (q, *J*_2-2’-1_=7.0, 2H, H-2), 1.87−1.82 (m, 1H, CH-1’), 2.12 (ddq, *J*_2’-3’a-2-_=7.1, *J*_2’-3’s_=11.0, *J*_2’-1’_=2.0, 1H, H-2’), 1.47 (ddt*, J*_3’a-3’s_=14.2, *J*_3’a-2’-4’s_=5.8, *J*_3’a-4’a_=11.1, 1H, H-3’a), 2.01−1.92 (m, 1H, H-3’s), 1.89−1.82 (m, 1H, H-4’a), 1.97−1.90 (m, 1H, H-4’s), 1.91−1.87 (m, 1H, H-5’), 0.90 (d, *J*_7’s-7’a_=9.5, 1H, H-7’s), 2.33 (ddt, *J*_7’a-7’s_=9.3, *J*_7’a-4’a_=2.0, *J*_7’a-1’-5’_=6.2, 1H, H-7’a), 1.19 (s*,* 3H, Me-6’), 1.01 (s*,* 3H, Me’-6’); ^13^C**-**NMR (100 MHz): δ 61.68 (C-1), 40.76 (C-2), 46.40 (C-1’), 37.48 (C-2’), 22.34 (C-3’), 26.43 (C-4’), 41.42 (C-5’), 38.69 (C-6’), 33.60 (C-7’), 28.16 (Me-6’), 23.22 (Me’-6’).

*(1S,2S,5S)-6,6-Dimethylbicyclo[3.1.1]hept-2-yl-ethanal* (**18**): a solution of dihydronopol (**17**, 907 mg, 5.4 mmol) in dry CH_2_Cl_2_ (8 mL) was added to a solution of PDC (3.05 g, 8.1 mmol) in CH_2_Cl_2_ (22 mL) at 25 °C and stirred for 20 h under argon. Then, the mixture was diluted with diethylether–hexane, filtered and the solvent evaporated under reduced pressure to afford **18** (672 mg, 75%) as a yellow-pale oil, [α]^25^_D_ = –19.3 (*c* 1.10, MeOH). The GC analysis (*t*_R_ 21.44) indicated the conversion was complete. IR (ν, cm^-1^): 2907 and 2713 (cyclohexane), 1725 (C=O), 1384 and 1367 (C(CH_3_)_2_); MS (*m/z*, %): 166 (M^+^, 1), 151 (M^+^−Me, 9), 148 (M^+^−H_2_O, 4), 133 (M^+^−H_2_O−Me, 12), 123 (M^+^−CH_2_CHO, 27), 107 (C_8_H_11_^+^, 33), 79 (C_6_H_7_^+^, 82); ^1^H-NMR (400 MHz): δ 0.90 (d, *J*_7’s-7’a_=9.8, 1H, H-7’s), 0.95 (s, 3H, Me-6’), 1.12 (s*,* 3H, Me’-6’), 1.37 (m, 1H, H-3’a), 1.99 (ddt, *J*_3’s-3’a_*=*14.6, *J*_3’s-4’a_*=*3.0, *J*_3’s-4’s-2’_*=*10.4, 1H, H-3’s), 1.74−1.79 (m, 1H, H-1’), 1.73−1.92 (m, 2H, H-4’), 1.91−1.89 (m*,* 1H, H-5’), 2.28 (ddt, *J*_7’a-7’s_=9.3, *J*_7’a-4’a_=2.0, *J*_7’a-1’-5’_=6.0, 1H, H-7’a), 2.44 (ddd, *J*_2A-1_=2.0, *J*_2A-2B_=16.3, *J*_2A-2’_=7.3, 1H, H-2A), 2.47 (ddd*, J*_2B-1_=2.0, *J*_2A-2B_=16.3, *J*_2B-2’_=7.3, 1H, H*-*2B), 2.58 (ddt*, J*_2’-2_=7.3, *J*_2’-3’s_=17.1, *J*_2’-1’_=2.3, 1H, H_2_-2’); ^13^C**-**NMR (100 MHz): δ 202.54 (C-1), 51.81 (C-2), 46.11 (C-1’), 34.83 (C-2’), 21.83 (C-3’), 25.95 (C-4’), 40.89 (C-5’), 38.46 (C-6’), 33.20 (C-7’), 27.73 (Me-6’), 22.96 (Me’-6’).

### 3.3. Aldol condensation of ***12−15*** and ***18*** with butanone to give ***19, 20, 22**, **24** and **27***

(a) With subsequent acidic catalyzed dehydration: a 6.0 M solution of starting aldehydes (**12−15** and **18**) in MeOH (1.0 mL, 6.0 mmol) was added dropwise to a stirred solution of butanone (1.73 g, 24.0 mmol) and KOH (15 mg, 0.25 mmol) in MeOH (1.5 mL) at 0 °C for 1 h. Then, the mixture was allowed to warm to room temperature and stirring was continued for a further 8 h. The reaction was quenched with a 1N aqueous solution of AcOH (100 mL), the solvent was then partially evaporated *in vacuo* and the resulting crude diluted with Et_2_O (25 mL) and washed with 1 N AcOH solution (25 mL) and brine (3×25 mL). The crude was dried over anhyd Na_2_SO_4_ and evaporated to yield a yellow residue, which was used in the next reaction without further purification. Then, a Dean−Stark apparatus was fitted to a flask containing a solution of the above aldol crude reaction and TsOH·H_2_O (40 mg, 0.2 mmol) in dry toluene (10 mL), and the mixture was refluxed for 90 min. The solution was allowed to cool down and washed with an aqueous saturated NaHCO_3_ solution (3×25 mL), 1N AcOH solution (25 mL) and brine (3×25 mL), dried over anhyd Na_2_SO_4_ and evaporated *in vacuo***.** The aldol condensations of butanone (a) with **12** afforded (*E*)-3,6-dimethylhept-3-en-2-one (**19**) in a 88% yield, (b) with **13** afforded (*E*)-3-methyldec-3-en-2-one (**20**) and (*Z*)-2-pentylnon-2-enal (**21**) in 71% and 24% yields, respectively, (c) with **14** afforded 1,1,3,4,6-pentamethyl-4a,5,6,7,8,8a-hexahydro-1*H*-isochromene (**23**) in a 91% yield, (d) with **15** afforded (*E*)-3-methyl-5-phenylpent-3-en-2-one (**24**) and its isomer (*E*)-3-methyl-5-phenylpent-4-en-2-one (**25**) in a 62% yield (**24**:**25,** 4:1) and (*E*)-2,4-diphenylbut-2-enal (**26**) in a 17% yield and (e) with **18** afforded (*E*)-3-methyl-5-((1*S*,2*S*,5S)-6,6-dimethylbicyclo[3.1.1]hept-2-yl)pent-3-en-2-one (**27**) in a 56% yield.

(b) With subsequent basic catalyzed dehydration**:** a solution of **14** (2.0 g, 12.9 mmol) in MeOH (4.0 mL) was added dropwise to a stirred solution of butanone (3.74 g, 51.9 mmol) and KOH (30 mg, 0.5 mmol) in MeOH (2.0 mL) at 0 °C for 1 h. Then, the mixture was allowed to warm to room temperature and stirring was continued for a further 8 h. A condenser was then fitted to the flask and the mixture heated at *ca*. 50 °C for 2 h. The solution was allowed to reach room temperature and quenched with 1N AcOH solution (25 mL). The mixture was extracted with Et_2_O (3×25 mL), and the combined organic extracts were neutralized by washing with brine (3×25 mL). The crude was dried over anhyd Na_2_SO_4_ and evaporated *in vacuo* to afford (*E*)-3,6,10-trimethylundeca-3,9-dien-2-one (**22**) in a 52% yield.

*(E)-3,6-Dimethylhept-3-en-2-one* (**19**): colourless oil; *t*_R_ 12.42; IR (ν, cm^-1^): 1671 and 1642 (α,β-unsaturated C=O), 1466 (-CH_2_-C=C-) and 1433 (CH_3_-C=C-); MS (*m/z*, %) 140 (M^+^, 14), 125 (M^+^−Me, 22), 97 (M^+^−[Me-C=O], 12), 83 (CH_3_-CO-CH-CH_3_^+^, 62), 69 (20), 55 (65), 43 (Me-C=O^+^, 100); ^1^H-NMR (300 MHz): δ 0.96 (d, *J*=6.6, 6H, H-7 and Me-6), 1.76 (br s, 3H, Me-3), 1.79 (n, *J*=6.7, 1H, H-6), 2.14 (t, *J*=7.1, 2H, H-5), 2.31 (*s*, 3H, H-1), 6.66 (tq, *J*_1_=7.4, *J*_2_=1.4, 1H, H-4); ^13C^ NMR (75 Hz): δ 11.03 (Me-3), 22.28 (C-7, Me-6), 25.20 (C-1), 28.20 (C-6), 37.97 (C-5), 137.98 (C-3), 142.47 (C-4), 199.54 (C-2).

*(E)-3-Methyldec-3-en-2-one* (**20**): the crude reaction mixture (**20**+**21**) was purified by flash chromatography (eluent: *n*-hexane/Et_2_O 95:5) to yield **20** as a yellow-pale oil; *t*_R_ 22.98. IR (ν, cm^-1^): 1671 and 1642 (α,β-unsaturated C=O), 1460 (-CH_2_-C=C-); MS (*m/z*, %): 168 (M^+^, 2), 153 (M^+^−Me, 8), 125 ( M^+^−[Me-C=O], 7), 111 (M^+^−[CH_3_-(CH_2_)_3_-], 4), 85 (M^+^−[Me-CO-C(Me)=CH], 14), 83 (M^+^−[CH_3_-(CH_2_)_5_-], 16), 69 (33), 55 (40), 43 (Me-C=O^+^, 100); ^1^H-NMR (300 MHz): δ 0.90 (t, *J*=6.7, 3H, H-10), 1.25-1.36 (m, 4H, H-7, H-8), 1.42-1.50 (m, 4H, H-6, H-9), 1.76 (br s, 3H, Me-3), 2.24 (q, *J*=7.3, 2H, H-5), 2.31 (s, 3H, H-1), 6.64 (tq, *J*_1_=7.3, *J*_2_=1.3, 1H, H-4); ^13^C-NMR (75 MHz): δ 11.02 (Me-3), 13.98 (C-10), 22.50 (C-9), 25.34 (C-1), 28.54 (C-5), 29.03 (C-7), 29.09 (C-6), 31.57 (C-8), 137.48 (C-3), 143.97 (C-4), 199.91 (C-2).

*1,1,3,4,6-Pentamethyl-4a,5,6,7,8,8a-hexahydro-1H-isochromene* (**23**): the crude reaction product was purified by flash chromatography (eluent: *n*-hexane) to yield **23** as a colourless oil; *t*_R_ 26.00; IR (ν, cm^‑1^): 1738_,_ 1715, 1677, 1456; MS (*m/z*, %): 208 (M^+^, 22), 193 (M^+^−Me, 8), 190 (M^+^−H_2_O, 1), 175 (M^+^−Me−H_2_O, 2), 165 (M^+^−[O-C(Me)], 27), 150 (M^+^−[O-C(Me)]−Me, 7), 137 ((M^+^+1)−[O-C(Me)=C(Me)], 12), 123 (24), 109 (38), 95 (18), 83 (C_6_H_11_^+^, 10), 81 (C_6_H_9_^+^, 20), 69 (C_5_H_9_^+^, 28), 55 ([C-O-C(Me)]^+^, 37), 43 ([O-C(Me)]^+^, 100), 41 (74); ^1^H-NMR (300 MHz): δ 0.59 (q, *J*_5ax-5eq-4a-6_=12, 1H, H-5ax), 0.80−1.00 (m*,* 1H*,* H-7ax), 0.91−1.00 (m*,* 1H*,* H-8ax),0.92 (d, *J*=6.6, 3H, Me-6), 1.00 (s, 3H, Me_ax_-1), 1.12−1.22 (m, 1H, H-8a), 1.22 (s, 3H, Me_eq_-1), 1.33−1.46 (m, 1H, H-6), 1.53 (br s, 3H, Me-4), 1.55−1.68 (m*,* 1H*,* H-4a), 1.63−1.72 (m*,* 1H*,* H-8eq), 1.63−1.72 (m*,* 1H*,* H-7eq), 1.71 (br s, 3H, Me-3), 1.98−2.05 (m*,* 1H*,* H-5eq); ^13^C-NMR (75 MHz): δ 14.05 (Me-4), 17.22 (Me-3), 19.20 (Me_ax_-1), 22.64 (Me-6), 27.68 (Me_eq_-1), 27.74 (C-8), 32.77 (C-6), 35.32 (C-7), 38.37 (C-4a), 38.75 (C-5), 48.47 (C-8a), 75.40 (C-1), 103.15 (C-4), 141.75 (C-3).

*(E)-3,6,10-Trimethylundeca-3,9-dien-2-one* (**22**): the crude reaction product was purified by flash chromatography (eluent: *n*-hexane/Et_2_O 95:5) to yield **22** as a yellow-pale oil; *t*_R_ 28.69; IR (ν, cm^-1^): 1672 and 1642 (α,β-unsaturated C=O), 1455−1439 (CH_3_-C=C-); MS (*m/z*, %): 208 (M^+^, 1), 193 (M^+^−Me, 3), 165 (M^+^−[Me-C=O], 9), 150 (3), 136 (5), 125 ([(Me)_2_C=CH-(CH_2_)_2_-CH(Me)-CH_2_-]^+^, 9), 123 (14), 109 (26), 97 ([-CH_2_-CH=C(Me)-CO-Me]^+^, 3), 95 (10), 83 ([(Me)_2_C=CH-(CH_2_)_2_-]^+^, [CH=C(Me)-CO-Me]^+^, 11), 81 (12), 69 ([(Me)_2_C=C-CH_2_-]^+^, 52), 55 ([(Me)_2_C=CH-]^+^, 30), 43 (Me-C=O^+^, 94), 41 (100); ^1^H-NMR (300 MHz): δ 0.93 (d, *J*=6.6, 3H, Me-6), 1.18–1,42 (m, 2H, H-7), 1.34–1.64 (m, 1H, H-6), 1.61 (br s, 3H, Me-10), 1.69 (br s, 3H, H-11), 1.77 (br s, 3H, Me-3), 1.90–2.28 (m, 4H, H-5, H-8), 2.31 (s, 3H, H-1), 5.09 (br t, *J*=7.0, 1H, H-9), 6.66 (br t, *J*=7.4, 1H, H-4); ^13^C-NMR (75 MHz): δ 11.20 (Me-3), 17.54 (Me-10), 19.59 (Me-6), 25.35 (C-1), 25.47 (C-8), 25.61 (C-11), 32.66 (C-6), 36.29 (C-7), 36.82 (C-5), 124.31 (C-9), 131.37 (C-10), 138.15 (C-3), 142.66 C-4), 199.70 (C-2).

*(E)-3-Methyl-5-phenylpent-3-en-2-one* (**24**) *and (E)-3-methyl-5-phenylpent-4-en-2-one* (**25**): the crude reaction product was purified by vacuum distillation (60 °C, 0.08 Torr) to yield a 4:1 mixture of **24** and **25** as a brown oil; *t*_R_ 29.80 (**24**) and 28.76 (**25**); IR (ν, cm^-1^): 3084, 3060, 3027 (C=C, Ar), 1694 (CH_3_COCH(CH_3_)C), 1694 and 1667 (C=C(CH_3_)COCH_3_), 1452 (CH_2_C=C); MS (*m/z*, %) of **24**: 175 (M^+^+1, 11), 174 (M^+^, 9), 159 (M^+^–Me, 29), 131 (M^+^–MeCO, 100), 91 (C_7_H_7_^+^, 99), 77 (C_6_H_5_^+^, 12). MS (*m/z*, %) of **25**: 174 (M^+^, 9), 131 (M^+^–MeCO, 100), 116 (M^+^–MeCO−Me, 15), 91 (C_7_H_7_^+^, 46), 77 (C_6_H_5_^+^, 8); ^1^H-NMR (400 MHz): δ 1.27 (d, *J*_Me-3-3_=7, 3H, Me-C-3, **25**), 1.89 (d, *J*_Me-3-4_=1.2, 3H, Me-C-3, **24**), 2.19 (s, 3H, Me-1, **25**), 2.30 (s, 3H, Me-1, **24**), 3.35 (q, *J*_3-4-Me-3_=7.5, 1H, H-3, **25**), 3.59 (d, *J*_4-5_=7.5, 2H, H-5, **24**), 3.59 (d, *J*_4-5_=7.5, 2H, H-4, **25**), 6.52 (d, *J*_5-4_=15.9, 1H, H-5, **25**), 6.76 (tq, *J*_4-5_=7.2, *J*_4-Me-3_=1.2_,_ 1H, H-4, **24**), 7.15−7.50 (m, 5H, Ph, **24** and**25**); ^13^C-NMR (100 MHz): δ 11.34 (Me-C3, **24**), 25.51 (C-1, **24**), 35.36 (C-5, **24**), 127.64 (C-4’,**24**), 128.48 (C-5’ and -3’, **24**), 128.76 (C-2’ and -6’, **24**), 138.07 (C-3, **24**), 138.92 (C-1’, **24**), 141.49 (C-4, **24**), 199.79 (C-2, **24**); 16.15 (Me-3, **25**), 28.14 (C-1, **25**), 51.34 (C-3, **25**), 126.24 (C-5’ and -3’, **25**), 127.46 (C-4’,**25**), 128.48 (C-2’ and -6’, **25**), 128.74 (C-4, **25**), 132.15 (C-5, **25**), 135.81 (C-1’, **25**), 208.08 (C-2, **25**).

*(E)-3-Methyl-5-((1S,2S,5S)-6,6-dimethylbicyclo[3.1.1]hept-2-yl)pent-3-en-2-one* (**27**): the crude reaction product was purified by flash chromatography (eluent: *n*-hexane/Et_2_O 4:1) to yield **27** as a colourless oil; [α]^25^_D_ = –16.3 (*c* 1.35, MeOH); *t*_R_ 38.06; IR (ν, cm^-1^): 2982 and 2907 (cyclohexane), 1669 and 1641 (CH=C(CH_3_)COCH_3_), 1468 and 1431 (CH=CCH_3_), 1365 and 1387 (C(CH_3_)_2_); MS (*m/z*, %): 220 (M^+^, 1), 205 (M^+^−Me, 9), 177 (M^+^−Me−CO, 13), 137 (M^+^−CH=C(CH_3_)COCH_3_, 7), 123 (M^+^ −CH_2_CH=C(CH_3_)COCH_3_, 27), 83 (CH=C(CH_3_)COCH_3_^+^, 20), 43 (CH_3_CO^+^, 86); ^1^H-NMR (400 MHz): δ 0.89 (d, *J*_7’s-7’a_=9.0, 1H, H-7’s), 1.07 (s, 3H, CH_3_-9’), 1.19 (s, 3H, CH_3_-8), 1.50 (ddt, *J*_3’a-3’s_=14.5, *J*_3’a-4’a_=11.5, *J*_3’a-4’s-2’_=5.9, 1H, H-3’a), 1.76 (q, *J*_Me-3-4_=1.1, 3H, CH_3_-3), 1.88–1.82 (m, 1H, H-1’), 1.95–1.89(m, 1H, H-2’), 1.91–1.85 (m, 1H, H-4’a), 2.00–1.91 (m, 1H, H-4’s), 2.04–1.93 (m, 1H, H-3’s), 2.19 (ddt, *J*_5’-1’_=2.0, *J*_5’-7’a_=2.6, *J*_5’-4’s-4’a_=7.4, 1H, H-5’), 2.29 (s, 3H, CH_3_-1), 2.39–2.32 (m, 1H, H-7’a), 2.35–2.30 (m, 2H, H-5), 6.62 (tq, *J*_4-5_=7.3 and *J*_4-Me–3_=1.3_,_ 1H, H-4); ^13^C-NMR (100 MHz): δ 11.36 (Me-C-3), 22.28 (C-3’), 23.18 (Me-9’), 25.43 (C-1), 26.36 (C-4’), 28.12 (Me-8’), 33.81 (C-7’), 36.78 (C-5), 38.71 (C-6’), 41.13 (C-5’), 41.33 (C-2’), 45.79 (C-1’), 137.83 (C-3), 143.41 (C-4), 199.92 (C-2).

### 3.4. Deconjugative α-methylation of ***19, 20, 22,24*** and ***27*** to give ***28−32***

A solution of the appropriate α,β-unsaturated ketone [**19, 20, 22,24** (+**25**) or **27**] (60.5 mmol) in dry DMF (4 mL) was added dropwise to a stirred solution of K^t^BuO (6.98 g, 61.0 mmol) in dry DMF (30 mL) at room temperature for 30 min. After the addition was completed, the reaction was stirred for 10 min and then cooled to 0 °C. Pre-cooled MeI ( 22.81 g, 160.7 mmol) was added quickly and the reaction mixture stirred at that temperature for 10 min and then allowed to warm to room temperature. Brine (10 mL) and 1N AcOH solution (10 mL) were added and the crude was extracted with hexane/Et_2_O 1:1 (75 mL). The resulting organic solution was washed with 1N AcOH solution (2×30 mL) and brine (3×30 mL), dried over anhyd Na_2_SO_4_ and evaporated under reduced pressure to afford crude β,γ-unsaturated ketones **28**–**32**, which were all purified by flash chromatography (eluent: hexane/Et_2_O). The deconjugative α-methylation reaction (a) of **19** afforded (*E)*-3,3,6-trimethylhept-4-en-2-one (**28**) in a 77% yield, (b) of **20** afforded (*E*)-3,3-dimethyldec-4-en-2-one (**29**) in a 42% yield, (c) of **22** afforded (*E*)-3,3,6,10-tetramethylundeca-4,9-dien-2-one (**30**) in a 55% yield, (d) of a 4:1 mixture of **24** and **25** afforded (*E*)-3,3-dimethyl-5-phenylpent-4-en-2-one (**31**) in a 84% yield, and (e) of **27** afforded (*E*)-3,3-dimethyl-5-((1*S*,2*S*,5*S*)-6,6-dimethylbicyclo[3.1.1]hept-2-yl)pent-4-en-2-one (**32**) in a 85% yield.

*(E)-3,3,6-Trimethylhept-4-en-2-one* (**28**): yellow-pale oil; IR (ν, cm^-1^): 1712 (C=O), 1672 (H-C=C-H) and 1467 (-CH-C=C-C-); MS (*m/z*, %): 154 (M^+^, 1), 111(M^+^−C(CH_3_)_2_, 51), 69 ((CH_3_)_2_CH-CH=CH-^+^, 100), 55 ((CH_3_)_2_CH-C-^+^, 46), 43 (CH_3_-C=O^+^, 51); ^1^H-NMR (300 MHz): δ 0.98 (d, *J*=6.9, 6H, Me-7 and Me-C6), 1.20 (s, 6H, 2 Me-C3), 2.09 (s, 3H, Me-1), 2.29 (o, *J*=6.3, 1H, H-6), 5.42 (d, *J*_4-5_=15.7, 1H, H-4), 5.50 (dd, *J*_5-4_=15.8, *J*_5-6_=5.6, 1H, H-5); ^13^C**-**NMR (75 MHz)**:** δ 22.35 (2 Me-C3), 24.01 (C-7 and Me-C6), 25.21 (C-1), 31.16 (C-6), 49.85 (C-3), 131.33 (C-5), 137.48 (C-4), 211.92 (C-2).

*(E)-3,3-Dimethyldec-4-en-2-one* (**29**): yellow-pale oil; IR (ν, cm^-1^): 1713 (C=O) and 1467 (-CH-CH=CH-C-); MS (*m/z*, %): 182 (M^+^, 1), 167 (M^+^−Me, 1), 139 (M^+^−Me-C=O, 17), 97 (CH_3_-(CH_2_)_4_-CH-^+^, 8), 85 (Me-CO-C(Me)_2_-^+^, 1), 83 (33), 69 (100), 57 (CH_3_-(CH_2_)-^+^, 4), 55 (28), 43 (Me-C=O^+^); ^1^H-NMR (300 MHz): δ 0.89 (t, *J*=6.9, 3H, Me-10), 1.20 (s, 6H, 2 Me-C3), 1.22–1.41 (m, 6H, H-7, H-8, H-9), 2.03 (q, *J*=6.5, 2H, H-6), 2.10 (s, 3H, Me-1), 5.46 (d*, J*=15.6, 1H, H-4), 5.55 (dd, *J*_5-4_=6.0, *J*_5-6_=15.6, 1H, H-5); ^13^C-NMR (75 MHz):δ 13.88 (C-10), 22.42 (C-9), 23.99 (2 Me-C-3), 25.27 (C-1), 28.94 (C-7), 31.28 (C-8), 32.62 (C-6), 50.06 (C-3), 130.53 (C-5), 134.16 (C-4), 211.92 (C-2).

*(E)-3,3,6,10-Tetramethylundeca-4,9-dien-2-one* (**30**): yellow-pale oil; IR (ν, cm^-1^): 1712 (C=O), 1677 and 1628 (C=C), 1455 (-CH-C=C-C-); MS (*m/z*, %): 222 (M^+^, 1), 207 (M^+^−Me, 1), 179 (M^+^−Me-C=O, 5), 137 (M^+^−(Me)_2_C-CO-Me, 2), 124 ((Me)_2_-C=C-(CH_2_)_2_-CH(Me)-CH^+^, 3), 123 (13), 109 ((Me)_2_-C=CH-(CH_2_)_2_-CH(Me)-CH^+^−Me, 23), 95 (12), 83 ((Me)_2_-C=CH-(CH_2_)_2_^+^, 32), 69 ((Me)_2_-C=CH-CH_2_^+^,100), 55 ((Me)_2_-C=CH^+^, 21), 43 (Me-C=O^+^, 54); ^1^H-NMR (300 MHz): δ 0.90 (d, *J*=6.6, 3H, Me-C-6), 1.13 (s, 6H, 2 Me-C-3), 1.17–1.29 (m, 2H, H-7), 1.51 (br s, 3H, Me-11), 1.61 (br s, 3H, Me-C-10), 1.83 (q, 2H, H-8), 2.03 (s, 3H, Me-1), 2.40 (q, *J*=7.4, 1H, H-6), 5.01 (br t, *J*=7.1, 1H, H-9), 5.30 (dd, *J*_5-4_=15.7, *J*_5-6_=6.8, 1H, H-5), 5.38 (d, *J*=15.7, 1H, H-4);^13^C**-**NMR (75 MHz):δ 17.62 (C-11), 20.63 (Me-C-6), 24.05 and 24.12 (2 Me-C-3), 25.30 (C-1), 25.57 (Me-C-10), 25.80 (C-8), 36.56 (C-6), 37.06 (C-7), 50.02 (C-3), 124.47 (C-9), 131.31 (C-10), 132.77 (C-5), 136.27 (C-4), 211.86 (C-2).

*(E)-3,3-Dimethyl-5-phenylpent-4-en-2-one* (**31**): brown oil; IR (ν, cm^-1^): 3082, 3059 and 3026 (C=C, Ar), 1690 (CH_3_COC(CH_3_)_2_), 1679 and 971 (HC=CH), 1363 and 1363 (C(CH_3_)_2_), 749 and 694 (Ph); MS (*m/z*, %): 188 (M^+^, 1), 173 (M^+^-CH_3_, 1), 145 (M^+^−CH_3_CO, 30), 131 (M^+^−Me−CH_3_CO, 5), 91 (C_7_H_7_^+^, 99), 77 (C_6_H_5_^+^, 4), 43 (CH_3_CO^+^, 12), 28 (CO^+^, 100); ^1^H-NMR (400 MHz): δ 1.32 (s, 6H, 2CH_3_-C-3), 2.13 (s, 3H, Me-1), 6.26 (d, *J*_4-5_=16.2_,_ 1H, H-4), 6.45 (d, *J*_5-4_=16.3, 1H, H-5), 7.21 (tt, *J*_4’-3’-5’_=7.2, *J*_4’-2’-6’_=1.5, 1H, H-4’), 7.29 (t, *J*_3’-2’-4’_=7.3, 2H, H-3’ and 5’), 7.35 (dd, *J*_2’-3’/6’-5’_=7.1, *J*_2’-4’/6’-4’_=1.3, 2H, H-2’ and 6’); ^13^C**-**NMR (100 MHz): δ 23.91 (2CH_3_-C-3), 25.46 (C-1), 50.36 (C-3), 126.18 (C-2’ and C-6’), 127.47 (C-4), 128.48 (C-3’ and C-5’), 129.29 (C-4’), 133.99 (C-5), 136.85 (C-1’), 210.60 (C-2).

*(E)-3,3-Dimethyl-5-((1S,2S,5S)-6,6-dimethylbicyclo[3.1.1]hept-2-yl)pent-4-en-2-one* (**32**): yellow-pale oil; [α]^25^_D_ = –22.3 (*c* 1.15, MeOH); *R*_t_ 35.85; IR (ν, cm^-1^): 2939 and 2909 (cyclohexane), 1710 (CH_3_COC(CH_3_)_2_), 1672 and 972 (HC=CH), 1383 and 1364 (C(CH_3_)_2_). MS (*m/z*, %): 234 (M^+^, 1), 219 (M^+^–Me, 1), 191 (M^+^−CH_3_CO, 23), 149 (M^+^−CH_3_COC(CH_3_)_2_, 15), 93 (C_7_H_9_^+^, 3), 69 (CH_3_COCHCH_3_^+^, 100), 43 (CH_3_CO^+^, 77); ^1^H-NMR (400 MHz): δ 2.08 (s, 3H, Me-1), 5.37 (dd, *J*_4-5_=15.8, *J*_4-2´_=1.6_,_ 1H, H-4), 5.67 (dd, *J*_5-4_=15.8, *J*_5-2’_=6.8, 1H, H-5), 1.91–1.99 (m, 1H, CH-1’), 2.73 (ddddd, *J*_2’-3’s_=10.5, *J*_2’-5_ =6.8, *J*_2’-1’_=6.0, *J*_2’-3’a_ =2.5, *J*_2’-4_=1.7, 1H, H-2’), 1.60 (dddd, *J*_3’a-3’s_=15.3, *J*_3’a-4’a_=10.5, *J*_3’a-2’_=6.0, *J*_3’a-4’s_=4.5, 1H, H-3’a), 1.93–2.02 (m, 1H, H-3’s), 1.81–1.92 (m, 1H, H-4’a), 1.92–2.00 (m, 1H, H-4’s), 1.86–1.96 (m, 1H, H-5’), 0.99 (d, *J*_7’s-7’a_=9.7, 1H, H-7’s), 2.32 (dddd, *J*_7’s-7’a_=9.7, *J*_7’s-1’_=6.6, *J*_7’s-5’_=5.7, *J*_7’s-4’a_=1.6, 1H, H-7’a), 1.19 (s, 3H, Me-8), 0.95 (s, 3H, Me-9), 1.19 (s, 6H, 2CH_3_-C-3); ^13^C**-**NMR (100 MHz): δ 21.42 (C-3’), 23.53 (Me-9’), 24.03 (2CH_3_-C-3), 25.35 (C-1), 26.00 (C-4’), 27.83 (Me-8’), 32.31 (C-7’), 38.53 (C-6’),41.08 (C-5’), 43.34 (C-2’), 46.99 (C-1’), 49.96 (C-3), 132.09 (C-4), 137.15 (C-5), 211.80 (C-2).

### 3.5. Reduction of ***28**–**32*** with NaBH_4_ to give ***33**–**37***

Solid NaBH_4_ (2.77 g, 71.8 mmol) was added portionwise to a stirred solution of starting β,γ-unsaturated ketone **28**–**32** (54.7 mmol) in MeOH (50 mL) at 0 °C. After 15 min the reaction was allowed to warm to rt and left to react for 45 min. Then, the solvent was partially evaporated under reduced pressure and the resulting suspension was diluted with hexane/Et_2_O 1:2 (75 mL), cooled again to 0 °C and neutralized with 1N AcOH solution. The organic layer was washed again with 1N AcOH solution (50 mL) and brine (3 × 50 mL), then dried over anhyd Na_2_SO_4_ and the solvent evaporated *in vacuo* to yield crude alcohols **33**–**37**, which were purified by flash chromatography (eluent: hexane/Et_2_O). The reduction with NaBH_4_ (a) of **28** afforded (*E*)-3,3,6-trimethylhept-4-en-2-ol (**33**) in a 62% yield, (b) of **29** afforded (*E*)-3,3-dimethyldec-4-en-2-ol (**34**) in a 76% yield, (c) of **30** afforded (*E*)-3,3,6,10-tetramethylundeca-4,9-dien-2-ol (**35**) in a 90% yield, (d) of **31** afforded (*E*)-3,3-dimethyl-5-phenylpent-4-en-2-ol (**36**) in a 80% yield, and (e) of **32** afforded (*E*)-3,3-dimethyl-5-((1*S*,2*S*,5*S*)-6,6-dimethylbicyclo[3.1.1]hept-2-yl)pent-4-en-2-ol (**37**) in a 77% yield.

*(E)-3,3,6-Trimethylhept-4-en-2-ol* (**33**): yellow-pale oil; *t*_R_ 11.62; IR (ν, cm^-1^): 3684 (OH), 1466 (CH-C=C-C); MS (*m/z*, %): 142 (M^+^−14, 1), 127 (1), 123 (M^+^−Me–H_2_O, 1), 113 (M^+^−(CH_3_)_2_CH, 2), 112 (25), 69 ((CH_3_)_2_CH-CH=CH^+^, 100), 56 ((CH_3_)_2_CH-CH=C^+^, 22), 55 (34), 43 ((CH_3_)_2_CH^+^, 38); ^1^H- NMR (300 MHz): δ 0.97 (s, 6H, 2CH_3_-C-3), 0.99 (d, *J*=6.6, 6H, Me-7 and Me-C-6), 1.09 (d, *J*=6.3, 3H, Me-1), 2.28 (o, *J*=6.7, 1H, H-6), 3.46 (q, *J*=6.4, 1H, H-2), 5.34 (d, *J*_4-5_=15.7, 1H, H-4), 5.43 (dd, *J*_5-4_=15.7, *J*_5-6_=6.2, 1H, H-5); ^13^C-NMR (75 MHz): δ 17.24 (C-1), 21.78*^a^ (Me-C-3), 22.79*^b^ (Me-C-6), 22.81*^b^ (Me-7), 24.03*^a^ (Me^’^-C-3), 31.35 (C-6), 40.44 (C-3), 74.13 (C-2), 133.55 (C-5), 136.98 (C-4) (*these signals may be interchanged); HRMS *m/z*, calcd. for C_10_H_20_O, 156.1514 (M^+^), found 156.1467.

*(E)-3,3-Dimethyldec-4-en-2-ol* (**34**): yellow-pale oil; *t*_R_ 22.25; IR (ν, cm^-1^): 3841−3400 (OH), 1467 (CH_2_-CH=CH); MS (*m/z*, %): 169 (M^+^−Me, 1), 167 (M^+^−OH, 1), 140 (13), 139 (Me-CH-OH^+^, 3), 125 (4), 112 (5), 97 (CH_3_-(CH_2_)_4_-CH=CH^+^, 11), 83 (31), 69 (100), 55 (34), 43 (27), 41 (50); ^1^H-NMR (300 MHz): δ 0.89 (t, *J*=6.5, 3H, Me-10), 0.97 (s, 6H, 2Me-C-3), 1.09 (d, *J*=6.3, 3H, Me-1), 1.25–1.44 (m, 6H, H-7, H-8, H-9), 2.02 (q, *J*=6.6, 2H, H-6), 3.46 (q, *J*=6.2, 1H, CH-2), 5.38 (d, *J*_4-5_=15.9, 1H, H-4), 5.47 (dd, *J*_5-6_=6.1, *J*_5-4_=15.7, 1H, H-5); ^13^C-NMR (75 MHz): δ 14.02 (C-10), 17.24 (C-1), 21.73*^a^ (Me-C-3), 22.47 (C-9), 24.03*^a^ (Me-C-3), 29.30 (C-7), 31.36*^b^ (C-8), 32.83*^b ^(C-6), 40.68 (C-3), 74.11 (C-2), 129.83 (C-5), 136.57 (C-4) (*these signals may be interchanged); HRMS *m/z*, calcd. for C_12_H_20_O, 184.1827 (M^+^), found 184.1183.

*(E)-3,3,6,10-Tetramethylundeca-4,9-dien-2-ol* (**35**): yellow-pale oil; *t*_R_ 31.25; IR (ν, cm^-1^): 3430 (OH) and 1455 (CH_2_-CH=CH); MS (*m/z*, %): 180 ((M^+^+1)−[Me-CH-OH], 1), 137 (M^+^−[(Me)_2_C-CHOH-Me], 8), 123 (19), 109 ([(Me)_2_-C=CH-(CH_2_)_2_-CH(Me)-CH]^+^−Me, 50), 95 (21), 83 ([(Me)_2_-C=CH-(CH_2_)_2_]^+^, 29), 69 ([(Me)_2_-C=CH-CH_2_]^+^,100), 55 ([(Me)_2_-C=CH]^+^, 30), 45 (54); ^1^H-NMR (300 MHz): δ 0.96–0.98 (m, 3H, Me-C-6), 0.98 (s, 6H, 2 Me-3), 1.09 (d, *J*=6.5, 3H, Me-1), 1.29 (q, *J*=7.4, 2H, CH_2_-7), 1.58 (br s, 3H, Me-C-10), 1.68 (br s, 3H, Me-11), 1.93 (q, *J*= 7.6, 2H, CH_2_-8), 2.11 (m, *J*=6.8, 1H, CH-6), 3.46 (q, *J*=6.2, 1H, CH-2), 5.09 (br t, *J*=7.2, 1H, CH-9), 5.29 (dd, *J*_1_=16.0, *J*_2_= 6.6, 1H, CH-5), 5.36 (d, *J*=15.6, 1H, CH-4); ^13^C-NMR (75 MHz): δ 17.28 (C-1), 17.62 (C-11), 21.04 (Me-C6), 21.97 (Me-C3), 23.98 (Me’-C3), 25.67 (Me-C10), 25.89 (C-8), 36.72 (C-6), 37.26 (C-7), 40.59 (C-3), 74.13 (C-2), 124.59 (C-9), 131.19 (C-10), 135.00 (C-5), 135.58 (C-4); HRMS *m/z*, calcd. for C_15_H_28_O, 224.2140 (M^+^), found 224.2157.

*(E)-3,3-Dimethyl-5-phenylpent-4-en-2-ol* (**36**): yellow oil; *t*_R_ 32.30; IR (ν, cm^-1^): 3406, 1093, 1071 (OH); 1383 (C(CH_3_)_2_); 1646, 972 (CH=CH). 1945, 1875, 1802, 911, 748, 693 (Ph); ^1^H-NMR (400 MHz): 1.10 (s, 6H, 2Me-C-3), 1.13 (*d*, *J*=6.4, 3H, Me-1), 3.58 (q, *J*=6.3, 1H, H-2), 6.22 (d, *J*_4-5_=16.3, 1H, H-4), 6.39 (*d*, *J*_5-4_=16.3, 1H, H-5), 7.19 (tt, *J*_4’-3’-5’_=7.2 and *J*_4’-2’-6’_=0.9, 1H, H-4’), 7.28 (dt, *J*_3’-2’-4’_=7.4 and *J*_3’-5’_=1.5, 2H, H-3’ and H-5’), 7.36 (br d, *J*_2’-3’_=7.4H, 2H, H-2’ and H-6’); ^13^C-NMR (100 MHz): 17.75 (C-1), 22.27 and 23.56 (2 M-C-3), 41.23 (C-3), 74.44 (C-2), 126.08 (C-2’ and C-6’), 127.08 (C-4), 128.41 (C-4’), 128.45 (C-3’ and C-5’), 136.94 (C-5), 137.48 (C-1’); HRMS *m/z*, calcd. for C_13_H_18_O, 190.1358 (M^+^), found 190.1345.

*(E)-3,3-Dimethyl-5-((1S,2S,5S)-6,6-dimethylbicyclo[3.1.1]hept-2-yl)pent-4-en-2-ol* (**37**): yellow-pale oil; [α]^25^_D_ = −20.9 (*c* 0.85, MeOH); *t*_R_ 36.70; IR (ν, cm^-1^): 3475–3300 (OH), 1090, 1069 (CH-OH), 1384, 1366 (C(CH_3_)_2_); 1654, 975 (CH=CH); ^1^H-NMR (400 MHz): δ 1.08 (d, *J*=6.4, 3H, H-1), 3.46 (q, *J*=6.4, 1H, H-2), 5.29 (dd, *J*_4-5_=15.8 and *J*_4-2´_=1.5_,_ 1H, H-4), 5.62 (dd, *J*_5-4_=15.8, *J*_5-2’_=7.0, 1H, H-5), 1.89–1.99 (m, 1H, H-1’), 2.73 (dtt, *J*_2’-1’-4_=1.1, *J*_2’-3’a-5_=6.0, *J*_2’-3’s_=11.8 1H, H-2’), 1.61 (ddt, *J*_3’a-4’a_=5.6, *J*_3’a-3’s_=10.2, *J*_3’a-2’-4’s_=10.0, 1H, H-3’a), 1.93–2.03 (m, 1H, H-3’s), 1.81–1.92 (m, 1H, H-4’a), 1.92–1.99 (m, 1H, H-4’s), 1.86–1.97 (m, 1H, H-5’), 0.99 (d, *J*_7’s-7’a_=9.9, 1H, H-7’s), 2.32 (dt, *J*_7’s-7’a_=8.8, J_7’a-1’-5’_=6.1, 1H, H-7’a), 1.19 (s, 3H, Me_s_-C-6), 0.97 (s, 3H, Me_s_-C-6), 0.97 (*s*, 3H, Me-C-3), 0.96 (*s*, 3H, Me^’^-C-3); ^13^C-NMR (100 MHz): δ 17.25 (C-1), 21.69 and 21.74 (Me^’^-C-3), 21.82 and 21.77 (C-3’), 23.59 (Me-9), 24.03 (Me-C-3), 26.09 (C-4’), 27.90 (Me-8), 32.43 (C-7’), 38.58 (C-6’), 40.54 (C-3), 41.13 (C-5’), 43.60 (C-2’), 47.48 and 47.35 (C-1’), 74.21 and 74.18 (C-2), 134.46 (C-4), 136.74 (C-5); HRMS *m/z*, calcd. for C_16_H_28_O, 236.2140 (M^+^), found 236.1993.

### 3.6. Sensory evaluation

*Direct smelling analysis*. Blotting paper strips were impregnated with compounds **33**–**37**, previously diluted with Et_2_O (25 mg/200 μL), and smelt by perfumers at that moment (after solvent evaporation), 3 h and 24 h later. The olfactory description in each session therefore corresponded to the top, heart and base notes, respectively.

*GC sniffing analysis*. Odour assessment of compounds **33**–**37** was achieved by a group of perfumers using a Hewlett-Packard Model 5890 Series II gas chromatograph equipped with a thermal conductivity detector (TCD) and handmade sniffing port. Separation was done with a 10% Carbowax 20M over Chromosorb W/AW 80−100 mesh packed column (1.8 m×6 mm OD×2.2 mm ID); injector temperature: 250 °C; detector temperature: 250 °C, oven temperature program: 60 °C (0 min) to 240 °C (20 min) at 4 °C/min. Sample size for each injection was approximately 1 μL in a 1:10 split mode.

## 4. Conclusions

The literature SOR data on the sandalwood olfactophore seem to point that the bulky hydrophobic moiety of odorants such as β-santalol (**1**) and campholenal derivatives **2**–**5** could be replaced by substructures of similar steric bulk. Thus, new five bulky moiety modified analogues **33**–**37** of the commercial sandalwood odorant Polysantol^®^ (**2**) have been synthesized. Starting from the aldehydes isovaleraldehyde (**12**), heptanal (**13**), citronellal (**14**), phenylacetaldehyde (**15**) and dihydronopal (**18**), and by an expeditious sequence of aldol condensation with butanone, deconjugative α-methylation of the resulting α,β-unsaturated ketones, and reduction of the corresponding β,γ-unsaturated ketones, the new five analogues were prepared in good yield. These compounds **33-37** were organoleptically evaluated and one of them (compound **37**) seemed to be of special interest due to its natural sandalwood scent, which means that the dihydronopyl group is able to mimic the bulky hydrophobic center C of the sandalwood olfactophore. The other synthesized alcohols do not seem to be of interest as odorants, although the branched-chain citronellal derivative **35** and the aromatic-ring phenylacetaldehyde derivative **36** have some sandalwood notes, at least according to the GC sniffing odour evaluation.
